# Cannabinoid Content and Label Accuracy of Hemp-Derived Topical Products Available Online and at National Retail Stores

**DOI:** 10.1001/jamanetworkopen.2022.23019

**Published:** 2022-07-20

**Authors:** Tory R. Spindle, Dennis J. Sholler, Edward J. Cone, Timothy P. Murphy, Mahmoud ElSohly, Ruth E. Winecker, Ronald R. Flegel, Marcel O. Bonn-Miller, Ryan Vandrey

**Affiliations:** 1 Behavioral Pharmacology Research Unit, Johns Hopkins University School of Medicine, Baltimore, Maryland; 2 ElSohly Laboratories Inc, Oxford, Mississippi; 3 RTI International, Research Triangle Park, North Carolina; 4 Substance Abuse and Mental Health Services Administration, Division of Workplace Programs, Rockville, Maryland; 5 Canopy Growth Corporation, Smiths Falls, Ontario, Canada

## Abstract

**Importance:**

Products containing cannabinoids such as cannabidiol (CBD) have proliferated since 2018, when the Agriculture Improvement Act removed hemp (ie, cannabis containing <0.3% Δ^9^-tetrahydrocannabinol [THC]) from the US controlled substances list. Topical cannabinoid products can be purchased nationwide at retail stores and over the internet, yet research on these products is scarce.

**Objective:**

To evaluate the cannabinoid content (ie, CBD and THC) and label accuracy of topical cannabinoid products and to quantify their therapeutic and nontherapeutic claims.

**Design, Setting, and Participants:**

Product inclusion criteria included designation as hemp products, intended for topical or transdermal application, and purported to contain cannabinoids (eg, CBD). All unique products available at each retail store were purchased. Online products were identified via Google using relevant keywords (eg, hemp or CBD topical). Various products (eg, lotions and patches) were purchased from retail stores (eg, pharmacies, grocery stores, and cosmetic or beauty stores) in Baltimore, Maryland, and online. Data analysis was performed from March to June 2022.

**Main Outcomes and Measures:**

Labeled and actual total amounts of CBD and THC, measured via gas chromatography–mass spectrometry. Therapeutic and nontherapeutic claims and references to the US Food and Drug Administration were quantified.

**Results:**

A total of 105 products were purchased, 45 from retail locations and 60 online. Of the 89 products that listed a total amount of CBD on the label, 18% (16 products) were overlabeled (ie, contained >10% less CBD than advertised), 58% (52 products) were underlabeled (ie, contained >10% more CBD than advertised), and 24% (21 products) were accurately labeled. The median (range) percentage deviation between the actual total amount of CBD and the labeled amount was 21% (−75% to 93%) for in-store products and 10% (−96% to 121%) for online products, indicating that products contained more CBD than advertised overall. THC was detected in 37 of 105 products (35%), although all contained less than 0.3% THC. Among the 37 THC-containing products, 4 (11%) were labeled as THC free, 14 (38%) indicated they contained less than 0.3% THC, and 19 (51%) did not reference THC on the label. Overall, 28% of products (29 products) made therapeutic claims, 14% (15 products) made cosmetic claims, and only 47% (49 products) noted that they were not Food and Drug Administration approved.

**Conclusions and Relevance:**

In a case series of topical cannabinoid products purchased online and at popular retail stores, products were often inaccurately labeled for CBD and many contained THC. These findings suggest that clinical studies are needed to determine whether topical cannabinoid products with THC can produce psychoactive effects or positive drug tests for cannabis.

## Key Points

**Question:**

Are hemp-derived topical cannabinoid products available online and at national retailers accurately labeled for cannabidiol (CBD) and Δ^9^-tetrahydrocannabinol (THC) content?

**Findings:**

In a case series of 105 topical cannabinoid products, 18% were overlabeled (>10% less CBD than advertised), 58% were underlabeled (>10% more CBD than advertised), and 24% were accurately labeled for CBD; THC was detected in 35% of products (all contained less than 0.3% THC, the legal limit for hemp). Products often made therapeutic or cosmetic claims.

**Meaning:**

These findings suggest that improved regulatory oversight of cannabis and hemp products is needed to ensure quality assurance, deter misleading health claims, and potentially prevent unwanted drug effects among consumers.

## Teaser

This case series study evaluates the cannabinoid content and label accuracy of topical cannabinoid products and quantifies their therapeutic and nontherapeutic claims.

## Introduction

In 2018, the Agriculture Improvement Act (ie, The Farm Bill) removed hemp (cannabis containing <0.3% Δ^9^-tetrahydrocannabinol [THC], the primary psychoactive cannabis constituent) and its derivative products from the US controlled substances list. Consequently, various products containing hemp-derived cannabinoids have proliferated in both illicit and licit cannabis markets.^1^ Products containing hemp-derived cannabidiol (CBD) as a key constituent have become particularly popular, largely because of the growing interest in the use of CBD among consumers as an alternative treatment for various therapeutic conditions.^2^ Importantly, to date, the US Food and Drug Administration (FDA) has approved CBD to treat rare epilepsy disorders only.

Cannabinoid products intended for topical application (eg, lotions, creams, and patches) have seen arguably the largest growth since the Agriculture Improvement Act was enacted. Oral and vaping cannabinoid products are often labeled inaccurately for CBD and/or THC content,^3,4^ highlighting the poor regulatory oversight of cannabis products. However, to our knowledge, no such research has been conducted on hemp-derived topical cannabinoid products, which are now available for purchase nationwide at major retail stores and online. Of note, topical CBD products are increasingly being used to treat various health conditions (eg, pain and inflammation) or for cosmetic purposes (eg, antiaging), despite limited clinical research to demonstrate efficacy.^5^ In this case series study, we investigated the cannabinoid content and label accuracy of topical cannabinoid products and determined whether they made therapeutic or nontherapeutic claims.

## Methods

Products were purchased from brick-and-mortar retail locations and online. To be included, products had to be classified as hemp products, intended for topical or transdermal application, and purported to contain cannabinoids (eg, CBD) according to the product packaging and descriptions. This study was exempt from ethics review and informed consent because it did not involve human participants, in accordance with 45 CFR §46. This manuscript follows the recommended reporting guideline for case series studies.^6^

A list of 15 diverse national retailers that carried these products was created (eg, grocery stores, pharmacies, cosmetic and beauty stores, and health and wellness stores). Research team members visited stores in the Baltimore, Maryland, area from this list and purchased all unique products meeting the inclusion criteria (purchasing occurred from July 10, 2020, to July 20, 2020). For online purchasing, internet searches were performed via Google (keywords included hemp/CBD topical, hemp/CBD transdermal, full-spectrum hemp, and CBD topicals). A list was compiled of unique topical cannabinoid products found in the first 50 links for each keyword. After excluding products already purchased from retail locations, approximately one-half of the remaining products were randomly selected for purchase (online purchasing occurred from August 4, 2020, to August 6, 2020).

Products were stored in a cool, dark, and dry environment before being sent for gas chromatography–mass spectrometry testing for THC, CBD, and various other cannabinoids (limit of detection, 0.01% or 0.1 mg/g; see the eAppendix in the Supplement for a full description of the analytical method). This report focuses only on THC and CBD; other cannabinoids were rarely detected and generally at trace concentrations if present.

### Data Presentation

Products were considered accurately labeled for CBD if the measured total amount of CBD in the entire product was within 10% of the labeled total amount, and inaccurately labeled if greater than 10% or less than 10% of the labeled total amount. The 10% accuracy threshold is consistent with prior comparable studies^3,4^ and established standards for US Pharmacopeia herbal products and emerging cannabis industry standards. Therapeutic and cosmetic claims and references to the FDA were quantified as either present or not present on the basis of the product labels.

### Statistical Analysis

Data analysis was performed from March to June 2022. Kruskal-Wallis tests (*H*) examined whether actual amounts of CBD and THC or label accuracy for CBD differed across product type or purchase source. Significance was set at 2-sided *P* < .05. Analyses were conducted in SPSS statistical software version 25 (IBM). Individual groups were compared via Bonferroni-corrected post hoc tests (Dunn test, *Z*) if the omnibus statistical test was significant.

## Results

A total of 105 unique products were purchased and tested. Forty-five products from 29 companies were purchased from 7 different stores, and 60 products from 39 companies were purchased online. The median labeled total amount of CBD was 220 mg (range, 45-750 mg) among in-store products and 250 mg (range, 10-2500 mg) among online products (Table 1). The median actual total amount of CBD was 283 mg (range, 50-791 mg) among in-store products and 198 mg (range, 0.5-2820 mg) among online products. Actual total CBD amounts differed significantly across product type (*H*_5_ = 17.1; *P* = .004); patches contained less CBD than all other product types (patch vs cream, *Z* = 3.5, *P* = .007; patch vs balm, *Z* = 3.4, *P* = .009; patch vs lotion, *Z* = 3.5, *P* = .008; patch vs salve, *Z* = 3.5, *P* = .008). Actual total CBD amounts did not differ between online and in-store products.

**Table 1.** Labeling Accuracy of CBD Stratified by Product Type and Purchase Location

Labeling accuracy and amounts of CBD	Retail store products (n = 45)							Online products (n = 60)						
	Lotion (n = 7)	Cream (n = 15)	Balm (n = 9)	Salve (n = 3)	Patch (n = 1)	Other (n = 10)^a^	Total	Lotion (n = 8)	Cream (n = 17)	Balm (n = 7)	Salve (n = 9)	Patch (n = 10)	Other (n = 9)^a^	Total
Labeling accuracy, No. of products^b^														
Underlabeled	4	10	2	2	0	8	26	5	6	4	1	6	4	26
Overlabeled	1	0	3	0	0	1	5	2	4	2	2	0	1	11
Accurately labeled	0	3	2	0	0	0	5	0	6	1	3	4	2	16
Label total amount of CBD, mg														
Mean (SD)	238 (173)	276 (196)	231 (141)	425 (106)	NA	181 (123)	246 (163)	504 (452)	553 (599)	421 (343)	486 (483)	63 (68)	260 (215)	389 (448)
Median (range)	200 (100 to 600)	250 (80 to 750)	215 (45 to 400)	425 (350 to 500)	NA	200 (50 to 400)	220 (45 to 750)	300 (30 to 1250)	500 (20 to 2500)	250 (100 to 1000)	500 (125 to 1500)	50 (10 to 240)	150 (50 to 600)	250 (10 to 2500)
Actual total amount of CBD, mg														
Mean (SD)	352 (240)	272 (170)	254 (197)	493 (73)	NA	225 (147)	285 (187)	539 (513)	613 (724)	586 (520)	542 (558)	42 (33)	366 (388)	450 (549)
Median (range)	233 (168 to 791)	306 (50 to 537)	162 (63 to 574)	493 (441 to 544)	NA	281 (54 to 456)	283 (50 to 791)	340 (23 to 1424)	500 (6 to 2820)	276 (125 to 1440)	533 (24 to 1667)	36 (0.5 to 97)	167 (56 to 1106)	198 (0.5 to 2820)
Deviation between labeled and actual CBD total amount, %														
Mean (SD)	50 (25)	9 (51)	13 (40)	22 (48)	NA	23 (17)	22 (39)	19 (61)	4 (46)	30 (30)	2 (39)	–16 (42)	27 (44)	8 (45)
Median (range)	54 (16 to 93)	22 (–75 to 82)	14 (–73 to 64)	22 (–12 to 56)	NA	16 (8 to 56)	21 (–75 to 93)	42 (–92 to 80)	13 (–93 to 70)	25 (–1 to 88)	8 (–80 to 45)	–3 (–96 to 31)	12 (–5 to 121)	10 (–96 to 121)

Abbreviations: CBD, cannabidiol; NA, not applicable.

^a^ Other includes roll-on products, gels, oils, and serums intended for topical application.

^b^ Of the 105 products tested, 89 had a CBD total amount (in milligrams) on the label; the remaining 16 products did not contain a specific CBD amount on the label (eg, products were often labeled “*x* mg of full-spectrum hemp extract”).

Eighty-nine products listed a total amount of CBD (in milligrams) on the label; of these, 18% (16 products) were overlabeled (contained less CBD than advertised), 58% (52 products) were underlabeled (contained more CBD than advertised), and 24% (21 products) were accurately labeled. Overall, actual total CBD amounts were higher than labeled total amounts for both in-store products (median percentage deviation, 21%; range, −75% to 93%) and online products (median percentage deviation, 10%; range, −96% to 121%) (Figure). CBD label accuracy (ie, percentage deviation between labeled and actual amounts) did not differ significantly across purchase source (online vs in-store) but did differ across product type (*H*_5_ = 13.6; *P* = .02). Post hoc tests revealed that lotions were more underlabeled compared with patches (*Z* = 3.5; *P* = .008) (eFigure in the Supplement). There were no significant differences between the other product types.

**Figure.**
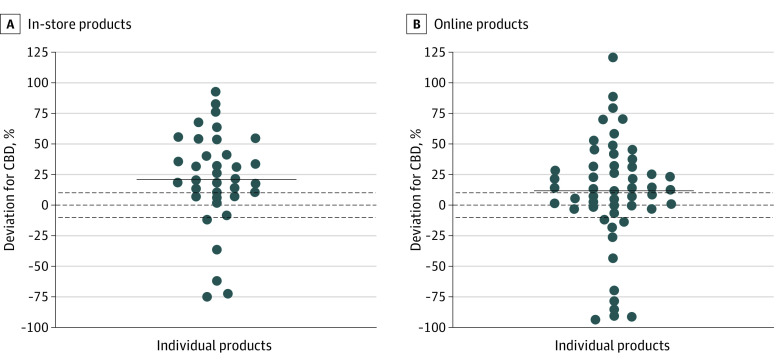
Percentage Deviation Between Actual and Labeled Amount of Cannabidiol (CBD) for Each Individual Product That Included an Amount of CBD (in Milligrams) on the Label, Stratified by Purchase Source Graphs show data for 89 products purchased in a store (A) and online (B). The solid lines depict the median percentage deviation. Zero (middle dashed line) indicates perfect agreement between actual and labeled CBD amounts, values greater than 10 (top dashed line) indicate the product contained more CBD than advertised (ie, underlabeled), and values less than −10 (bottom dashed line) indicate the product contained less CBD than advertised (ie, overlabeled). Each circle corresponds to an individual product.

THC was detected in 10 of 45 in-store products (22%) (median total amount of THC, 8 mg; range, 4-20 mg) and 27 of 60 online products (45%) (median total amount of THC, 16 mg; range, 0.03-100 mg) (Table 2). Of the 37 THC-containing products, 4 (11%) were labeled as THC free, 14 (38%) stated they contained less than 0.3% THC, and 19 (51%) did not reference THC on the label (Table 2). Actual total THC amounts were higher for online vs in-store products (*H*_1_ = 6.7; *P* = .01) but did not differ significantly across product types.

**Table 2.** Actual THC Concentration and Total Amount Stratified by Purported THC Content on Label and Purchase Location

Purchase location and THC content on label	Contained THC, products, No. (%)	Actual THC concentration across all products, %		Actual total THC amount across all products, mg		Actual total THC amount across THC-containing products, mg	
		Mean (SD)	Median (range)	Mean (SD)	Median (range)	Mean (SD)	Median (range)
Retail store products							
THC free (n = 16)	1 (6)	0.002 (0.010)	0.00 (0.00-0.03)	0.4 (1.8)	0.0 (0.0-7.1)	7.1 (NA)	7.1 (NA)
<0.3% THC (n = 11)	3 (27)	0.01 (0.02)	0.00 (0.00-0.05)	2.4 (4.6)	0.0 (0.0-14.2)	8.8 (4.7)	6.5 (5.8-14.2)
No mention of THC (n = 18)	6 (33)	0.01 (0.02)	0.00 (0.00-0.06)	3.3 (5.7)	0.0 (0.0-20.1)	9.9 (5.7)	9.1 (4.0-20.1)
Total (n = 45)	10 (22)	0.01 (0.02)	0.00 (0.00-0.06)	2.1 (4.5)	0.0 (0.0-20.1)	9.3 (4.9)	7.9 (4.0-20.1)
Online products							
THC free (n = 9)	3 (33)	0.03 (0.07)	0.00 (0.00-0.21)	0.1 (0.2)	0.0 (0.0-0.5)	0.3 (0.3)	0.40 (0.03-0.50)
<0.3% THC (n = 17)	11 (65)	0.03 (0.04)	0.03 (0.00-0.14)	11.9 (13.6)	8.2 (0.0-42.6)	18.4 (12.8)	15.5 (3.5-42.6)
No mention of THC (n = 34)	13 (38)	0.03 (0.05)	0.00 (0.00-0.22)	10.7 (22.2)	0.0 (0.0-100.2)	27.9 (28.8)	25.50 (0.08-100.20)
Total (n = 60)	27 (45)	0.03 (0.05)	0.00 (0.00-0.22)	9.4 (18.5)	0.0 (0.0-100.2)	21.0 (22.8)	15.50 (0.03-100.20)

Abbreviation: THC, Δ^9^-tetrahydrocannabinol.

Twenty-nine of 105 products (28%) made a therapeutic claim, most of which (22%) were about pain and inflammation (eTable in the Supplement). Fifteen products (14%) made a cosmetic or beauty claim (eg, alleviates wrinkles or nourishes and improves skin). Forty-nine products (47%) noted they were not FDA approved (eg, statements not evaluated by FDA); the remaining products made no reference to the FDA.

## Discussion

The market for hemp-derived topical cannabinoid products has seen substantial growth since 2018, but research on these products is lacking. This case series study tested topical cannabinoid products representative of those available throughout the US for CBD and THC content. Although there are few to no clinical data to inform whether commercial topical cannabinoid products such as those examined here can deliver THC and CBD to users, preclinical studies^7,8,9,10^ and clinical research with a pharmaceutical-grade CBD gel^11,12^ have shown that cannabinoids can penetrate the skin and enter systemic circulation when applied topically via some formulations. Thus, our findings may have important implications for consumers of topical cannabinoid products.

Most products were inaccurately labeled for CBD. Products with lower CBD than advertised may be less likely to elicit the desired medical benefits. Products with more CBD than advertised may be a health concern given the potential for adverse effects from CBD (eg, liver toxicity) and drug-drug interactions between CBD and common prescription medications.^13,14^ The labeled amount of CBD was usually expressed on the basis of the total contents of the product (eg, full lotion bottle) as opposed to the dose intended for each application. This labeling practice, in combination with vague or unclear application instructions, may contribute to inconsistent dosing and confusion among consumers.

THC was detected in 35% of products, most of which did not mention THC on the label or claimed to be THC free. Although THC concentrations were always under the legal limit for hemp (<0.3%), many products contained amounts of THC (up to 100 mg) capable of producing substantial intoxication when administered through other routes (eg, oral, smoked, or vaporized). That said, we measured the total amount of THC in these products and it is unlikely that someone would use an entire container on a single occasion. Controlled research is needed to determine whether topical products similar to those examined here can deliver THC to humans at levels capable of producing intoxicating effects. Research is also needed to determine whether topical products with THC can produce positive drug tests for cannabis, particularly when used repeatedly, as has been observed for hemp-based oral CBD products with low amounts of THC.^15^ Such research can inform whether individuals should abstain from using these products if they are drug tested for their occupation or other reasons. Ideally, future clinical studies should investigate a variety of topical products to elucidate whether product formulation is associated with cannabinoid absorption or acute drug effects.

The products examined often included therapeutic or cosmetic claims largely unsubstantiated by empirical evidence. For example, 22% of products claimed to be effective for pain relief, yet the FDA has not approved cannabinoid products (including topicals) for pain. Misleading claims may result in individuals using poorly regulated and expensive cannabinoid products instead of FDA-approved products that are established as safe and effective for a given health condition.

### Limitations

Study limitations include the purchasing of in-store products from 1 geographical location and online products via 1 search engine. In addition, we did not test for other potential contaminants that have relevance for safety (eg, pesticides and residual solvents).

## Conclusions

In conclusion, this case series found that topical cannabinoid products purchased online and at popular retail stores were often inaccurately labeled for CBD content and many contained the psychoactive cannabis constituent THC. Moreover, some products made therapeutic claims for indications not approved by the FDA. These findings highlight the need for proper regulatory oversight of cannabis and hemp products to ensure these products meet established standards for quality assurance and so that consumers are not misled by unproven therapeutic or cosmetic claims. These data also suggest that clinical studies are warranted to determine whether topical products with THC can produce psychoactive effects.

## Notes

Supplement. **eAppendix. **Analytical Methods **eTable.** Therapeutic and Nontherapeutic (ie, Cosmetic) Claims Based on Purchase Location **eFigure.** Percentage Deviation Between Actual and Labeled Amount of CBD for Each Individual Product That Included an Amount of CBD (in mg) on the Label (N=89), Stratified by Product Type
