# Supplementary material for: Cannabinoid Content and Label Accuracy of Hemp-Derived Topical Products Available Online and at National Retail Stores
Source: JAMA Netw Open. 2022 Jul 20;5(7):e2223019. doi: 10.1001/jamanetworkopen.2022.23019 (PMC9301515; doi:10.1001/jamanetworkopen.2022.23019)
Supplement: Supplement. — eAppendix. Analytical Methods eTable. Therapeutic and Nontherapeutic (ie, Cosmetic) Claims Based on Purchase Location eFigure. Percentage Deviation Between Actual and Labeled Amount of CBD for Each Individual Product That Included an Amount of CBD (in mg) on the Label (N=89), Stratified by Product Type [file jamanetwopen-e2223019-s001.pdf]

Supplemental Online Content

Spindle TR, Sholler DJ, Cone EJ, et al. Cannabinoid content and label accuracy of hemp-derived topical products available online and at national retail stores. *JAMA Netw Open*. 2022;5(7):e2223019. doi:10.1001/jamanetworkopen.2022.23019

eAppendix. Analytical Methods
eTable. Therapeutic and Nontherapeutic (ie, Cosmetic) Claims Based on Purchase Location
eFigure. Percentage Deviation Between Actual and Labeled Amount of CBD for Each Individual Product That Included an Amount of CBD (in mg) on the Label (N=89), Stratified by Product Type

This supplemental material has been provided by the authors to give readers additional information about their work.

© 2022 Spindle TR et al. *JAMA Network Open.*

eAppendix. Analytical Methods

GC/MS analysis for the presence of cannabinoids (i.e., THC, THC-A, CBD, and CBD-A, delta8-THC, CBC, CBDV, THCV, CBG, CBG-A, CBN) was carried out on the derivatized solutions of the tested products in the SIM mode utilizing d3 THC and d3 CBD internal standards. The limit of detection for all cannabinoids was 0.01% or 0.1mg/g. The analysis was performed on an Agilent Technologies 7890 A gas chromatograph with an Agilent Technologies 5975 C MSD and an Agilent Technologies 7693 autosampler. Separation was achieved using an Agilent Technologies 10M x 0.180 mm DB-1 column (0.40 film). Helium was used as the carrier gas at a flow rate of 0.5 mL/minute and the inlet was configured in splitless mode at a temperature of 250° C. The temperature program started at 180° C for one minute and then ramped up at 15° C/minute to 280° C for 2.3 minutes then 20° C/minute to 300° C for 2 minutes. The total run time was approximately 13 minutes. Data acquisition was performed on Enhanced ChemStation G1701EA.

© 2022 Spindle TR et al. *JAMA Network Open.*

eTable. Therapeutic and Nontherapeutic (ie, Cosmetic) Claims Based on Purchase Location.

Purchase Source	Retail Store Products (Total N=45)	Online Products (N=60)	Total (N=105)
Label Claim			
**No Claims**, # and %	27 (60%)	34 (56%)	61 (58%)
**Cosmetic (e.g., Beauty)**, # and %	8 (18%)	7 (12%)	15 (14%)
**Therapeutic**, # and %	10 (22%)	19 (32%)	29 (28%)
Pain/Inflammation, # and %	9 (20%)	14 (23%)	23 (22%)
Other[a], # and %	1 (2%)	5 (8%)	6 (6%)
**Stated Not FDA-approved on the Label**, # and %	16 (36%)	33 (55%)	49 (47%)

Note: [a] "Other" medical indications listed on labels included: anxiety, tattoo healing, sunburn prevention or treatment, and premenstrual cramps.

© 2022 Spindle TR et al. *JAMA Network Open.*

eFigure

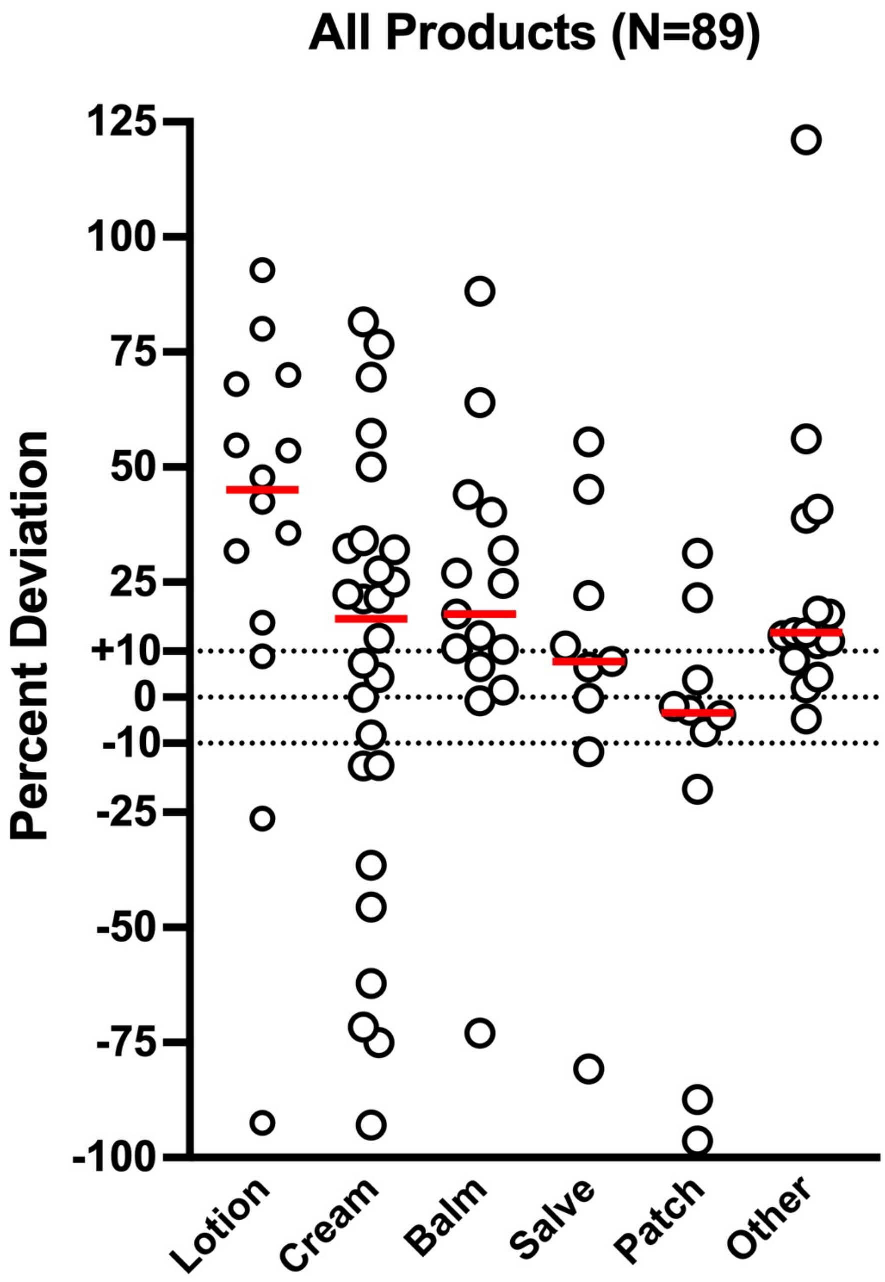

eFigure. Percentage Deviation Between Actual and Labeled Amount of CBD for Each Individual Product That Included an Amount of CBD (in mg) on the Label (N=89), Stratified by Product Type. The red lines depict the median percent deviation. Zero indicates perfect agreement between actual and labeled CBD amounts, values > +10 indicate the product contained more CBD than advertised (i.e., under-labeled), and values < -10 indicate the product contained less CBD than advertised (i.e., over-labeled).

© 2022 Spindle TR et al. *JAMA Network Open.*
